# Dissecting the impact of bromodomain inhibitors on the Interferon Regulatory Factor 4‐MYC oncogenic axis in multiple myeloma

**DOI:** 10.1002/hon.3016

**Published:** 2022-05-18

**Authors:** Alessandro Agnarelli, Simon Mitchell, Gillian Caalim, C. David Wood, Leanne Milton‐Harris, Timothy Chevassut, Michelle J. West, Erika J. Mancini

**Affiliations:** ^1^ Biochemistry and Biomedicine School of Life Sciences University of Sussex Brighton UK; ^2^ Brighton and Sussex Medical School University of Sussex Brighton UK

**Keywords:** BET/BRD4, CBP/EP300, dual inhibition, IRF4, multiple myeloma, MYC

## Abstract

B‐cell progenitor fate determinant interferon regulatory factor 4 (IRF4) exerts key roles in the pathogenesis and progression of multiple myeloma (MM), a currently incurable plasma cell malignancy. Aberrant expression of IRF4 and the establishment of a positive auto‐regulatory loop with oncogene MYC, drives a MM specific gene‐expression program leading to the abnormal expansion of malignant immature plasma cells. Targeting the IRF4‐MYC oncogenic loop has the potential to provide a selective and effective therapy for MM. Here we evaluate the use of bromodomain inhibitors to target the IRF4‐MYC axis through combined inhibition of their known epigenetic regulators, BRD4 and CBP/EP300. Although all inhibitors induced cell death, we found no synergistic effect of targeting both of these regulators on the viability of MM cell‐lines. Importantly, for all inhibitors over a time period up to 72 h, we detected reduced IRF4 mRNA, but a limited decrease in IRF4 protein expression or mRNA levels of downstream target genes. This indicates that inhibitor‐induced loss of cell viability is not mediated through reduced IRF4 protein expression, as previously proposed. Further analysis revealed a long half‐life of IRF4 protein in MM cells. In support of our experimental observations, gene network modeling of MM suggests that bromodomain inhibition is exerted primarily through MYC and not IRF4. These findings suggest that despite the autofeedback positive regulatory loop between IRF4 and MYC, bromodomain inhibitors are not effective at targeting IRF4 in MM and that novel therapeutic strategies should focus on the direct inhibition or degradation of IRF4.

## 1 INTRODUCTION

Transcription factor interferon regulatory factor 4 (IRF4) is a key activator of lymphocyte development, affinity maturation and terminal differentiation into immunoglobulin‐secreting plasma cells.^1^, ^2^ Faulty regulation of IRF4 expression is associated with numerous lymphoid malignancies, including multiple myeloma (MM), an aggressive and incurable hematologic cancer characterized by the abnormal proliferation of bone marrow plasma cells.^2^, ^3^ At the molecular level MM is an heterogenous disease with several subgroups defined by specific gene‐expression profiles and recurrent chromosomal rearrangements. In a minority of MM cases, chromosomal translocation t(6; 14) (p25; q32) brings the *IRF4* gene under the control of immunoglobulin heavy‐chain regulatory regions.^4^, ^5^ Interestingly while IRF4 is not always genetically altered in MM,^6^ its expression levels are always higher than in plasma cells.^7^ Over‐expression of IRF4 leads to an aberrant gene‐expression program and to the mis‐regulated transcription of a wide network of target genes. Interferon regulatory factor 4 loss‐of‐function in RNA‐interference‐based experiments have shown that MM cells are “addicted” to this abnormal gene‐expression program since reduced IRF4 expression causes rapid and extended non‐apoptotic cell death, irrespective of genetic etiology.^6^ Similarly, targeting the 3′ UTR of IRF4 mRNA for degradation by overexpression of miR‐125‐b, leads to MM cell death.^8^

MM accounts for 2% of all cancers and 10% of all hematologic malignancies.^9^ In the UK around 5800 MM cases are diagnosed every year (2015–2017) and incidence rates are projected to rise by 11% by 2035. The past decade has seen a revolution in the management of MM with the availability of novel therapies which are both more effective and less toxic. Despite the ensuing improvement of clinical outcomes, nearly every patient becomes refractory to therapies and overall 5‐year survival rates are 52%.^10^ Considering that existing treatments are not curative, there is a need for new therapeutic approaches. Targeting IRF4 has potential to be a powerful therapeutic strategy in MM. Firstly, IRF4 inhibition likely presents manageable side effects as phenotypes in IRF4‐deficient mice are restricted to lymphoid and myeloid lineages and mice lacking one allele of IRF4 are phenotypically normal.^6^ Additionally, MM cells' “addiction” to IRF4 renders them fairly sensitive to even small decreases in IRF4 levels leading to cell death. Finally, IRF4 inhibition is lethal to all MM cells regardless of their underlying transforming oncogenic mechanism.^6^

An attractive approach to inhibit IRF4 might be targeting a known regulator of IRF4 expression in MM, MYC. Constitutive activation of MYC signaling is detected in more than 60% of patient‐derived cells and one of the most common somatic genomic aberrations in MM is rearrangement or translocation of MYC.^11^ MYC transactivates *IRF4* by binding to a conserved intronic region whilst IRF4 binds to the *MYC* promoter region in MM cells and transactivates its expression, creating a positive autoregulatory feedback loop.^6^ The expression of MYC in MM cells is abnormal since normal plasma cells do not express MYC as a result of repression by PR domain zinc finger protein 1 (PRDM1).^12^ Moreover, IRF4 binds to its own promoter region, creating a second positive autoregulatory loop which would potentiate any therapeutic effect of targeting the MYC‐IRF4 loop.^6^ The IRF4‐MYC axis is thus considered to be a promising therapeutic target in MM, however the complex regulatory feedbacks make predictable targeting of this axis challenging.

One way to target the IRF4‐MYC axis is through upstream epigenetic regulators. Bromodomain and extra‐terminal (BET) proteins inhibitors have emerged as potential therapeutic agents for the treatment of hematologic malignancies.^13^ BET protein BRD4 is specifically enriched at immunoglobulin heavy chain (IgH) enhancers in MM cells bearing IgH rearrangement at the *MYC* locus, causing their aberrant proliferation.^14^ BET inhibitors such as JQ1, which displace BRD4 from chromatin by competitively binding to its bromodomain acetyl‐lysine recognition pocket, trigger inhibition of *MYC* transcription.^14^, ^15^

CREB binding protein (CBP) and EP300 are bromodomain‐containing histone acetyltransferases.^16^ CBP/EP300 bromodomain inhibitors, such as SGC‐CBP30, induce cell cycle arrest and apoptosis in MM cell‐lines.^17^ Whilst the effects of BET bromodomain inhibition are most likely due to direct suppression of MYC, inhibition of CBP/EP300 bromodomain has been proposed to work through suppression of IRF4.^17^

Given the positive auto regulation loop between MYC and IRF4 in MM, we hypothesized that combining the two classes of inhibitors with distinct transcriptional effects would have a synergistic impact on MM cells. To confirm this, we explored the effect of combinations of BET and CBP/EP300 inhibitors on the viability of a panel of MM cell‐lines. To assess whether the protein and mRNA levels for MYC, IRF4 and their downstream targets following drug exposure were consistent with those expected from the IRF4‐MYC auto‐regulatory loop model, we compared their experimentally measured with their simulated expression in a network model of MM molecular interactions. We found that within the time frames used there is no synergistic effect on the viability of MM cell‐lines. For all inhibitors we experimentally measured largely unaffected levels of IRF4 protein and downstream target protein mRNA levels. These results are consistent with the continued presence of IRF4 protein in MM cells due to its long half‐life. Our network modeling of MM therefore suggests that cell death induced by CBP/EP300 bromodomain inhibition is not exerted directly through IRF4 but indirectly through MYC.

## 2 METHODS

### 2.1 Cell viability assay

Cell viability assay and statistical analysis were performed as described in the supplemental methods. In brief, cell viability after inhibitors treatment was assessed using CellTiter‐Blue^®^ Cell Viability Assay. Each experiment was reproduced 3 times per cell line.

### 2.2 Western blotting

Detailed protocols for western blotting are available in the supplemental methods. Primary antibodies: IRF4 (ab133590, Abcam), MYC (sc‐40, Santa‐Cruz Biotechnology) and *β*‐actin (A2066, Sigma‐Aldrich). HRP‐conjugated secondary antibodies: anti‐rabbit (ab205718, Abcam) anti‐mouse (7076S, Cell signaling).

### 2.3 Quantitative real time PCR

RNA extraction, cDNA synthesis, and quantitative real time PCR was performed as in the supplemental methods.

### 2.4 Protein half‐life

To measure protein half‐life, cells were treated with 10 μg/ml cycloheximide for up to 72 h followed by western blotting. Detailed protocols are available in the supplemental methods.

### 2.5 Gene and protein network modeling

Computational models were constructed using Ordinary Differential Equations and solved using MATLAB 2020a and ode15 s. All code, equations and parameters used in modeling are available on Github (https://github.com/SiFTW/MMModel/). Regulated reactions were modeled as described previously.^18^ Detailed methods are available in the supplemental methods.

## 3 RESULTS

### 3.1 Concomitant BRD4 and CBP/EP300 inhibition does not have a synergistic effect on MM cell viability

**FIGURE 1:**
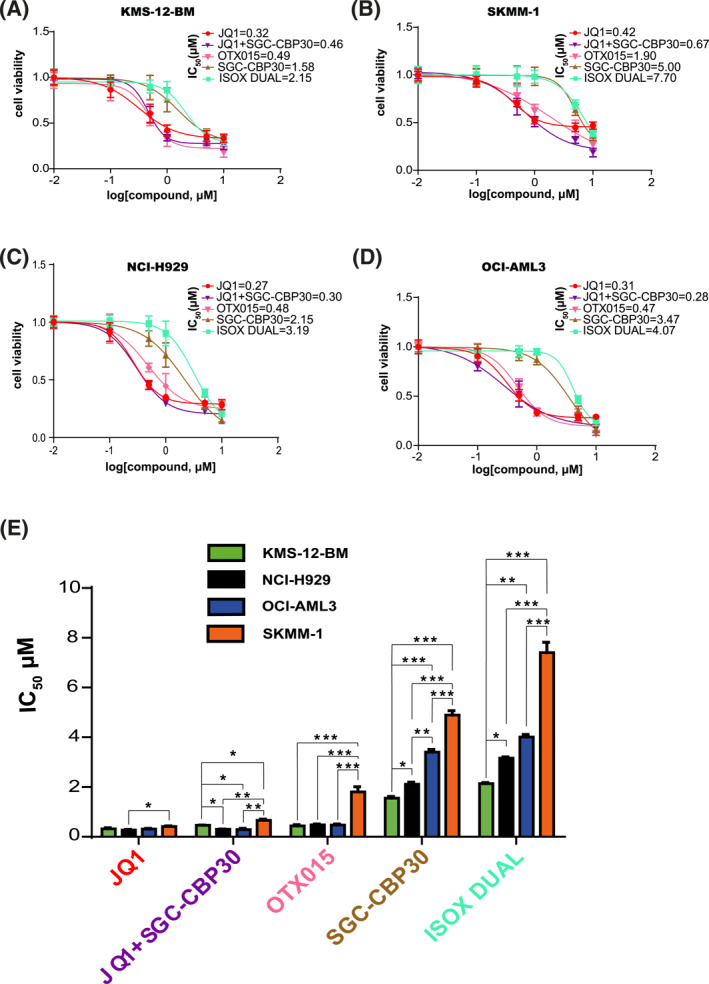
Characterization of the effect of JQ1, OTX015, SGC‐CBP30, ISOX‐DUAL and JQ1+ SGC‐CBP30 treatments on MM cell‐lines viability. Reduction of KMS‐12‐BM (A), NCI‐H929 (B), SKMM‐1 (C) and OCI‐AML3 (D) cell viability after treatment with different concentrations of bromodomain inhibitors for 48 h. Cell survival is plotted against the logarithm of inhibitor concentrations. JQ1 (red curves), JQ1+SGC‐CBPEP30 (purple curves), OTX015 (pink curves), SGC‐CBP30 (brown curves) and ISOX‐DUAL (light blue curves). Results are represented as mean ± Standard Error of Mean (SEM) of triplicate assays. (E) The graph shows the IC_50_ values of JQ1, JQ1+SGC‐CBP30, OTX015, SGC‐CBP/EP30, ISOX‐DUAL after 48 h treatment of KMS‐12‐BM (green bars), NCI‐H929 (black bars), OCI‐AML3 (blue bars) and SKMM‐1 (orange bars) cells

To explore the effect of the combination of bromodomain inhibitors on MM cell viability, we employed BET inhibitors JQ1 and OTX015, CPB/EP300 inhibitor SGC‐CBP30 and ISOX‐DUAL, a dual inhibitor of BET and CPB/EP300. Three MM (KMS‐12‐BM, NCI‐H929, SKMM‐1) and one acute leukemia (OCI‐AML3) cells lines were treated for 48 h with different concentrations of these compounds. As shown in Figure 1A–E, JQ1 was the most effective inhibitor with an IC_50_ between 0.27 and 0.42μΜ. Similar IC_50_ values were obtained for OTX015 (0.47–1.9 µM) and JQ1+SGC‐CBP30 (0.28–0.67 μM). However, treatment with SGC‐CBP30 alone (IC_50_ 1.58–5 μM) and ISOX‐DUAL (2.15–7.70 μM) showed reduced efficacy. The poor inhibitory activity of ISOX‐DUAL could be explained by its reduced affinity for BRD4 and CPB/EP300 (IC_50_ 1.5 and 0.65 μM) when compared to JQ1 and SGC‐CBP30.^19^ To test this hypothesis, we compared the effect of ISOX‐DUAL treatment with a combination of JQ1+SGC‐CBP30 (Figure 1E). We found that the combination treatment had a stronger inhibitory effect on cell viability than ISOX‐DUAL, with an IC_50_ comparable with that of JQ1 alone. Similar results were obtained when treating the cells for 72 h (Figure S1). Taken together, our results demonstrate that ISOX‐DUAL offers no advantage to treatment with a BET inhibitor alone and that combining JQ1 and SGC‐CBP30 does not lead to synergistic or antagonistic cytotoxic effects.

### 3.2 Bromodomain inhibitors impact IRF4 mRNA but not protein expression in MM cell‐lines

**FIGURE 2:**
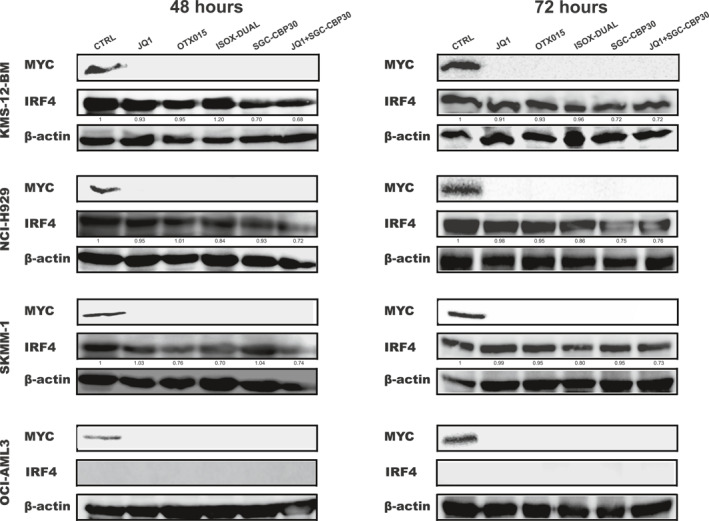
IRF4 and MYC protein levels in MM cell‐lines following treatment with JQ1, OTX015, SGC‐CBP30, ISOX‐DUAL and JQ1+ SGC‐CBP30. Changes in MYC and IRF4 protein levels were analyzed by Western Blot following IC_50_ drug treatments for 48 and 72 h in KMS‐12‐BM, SKMM‐1, NCI‐H929 and OCI‐AML3. The control (CTRL) is 2 mM DMSO treatment. *β*‐actin was used as loading control. Quantification was performed by using LI‐COR machine and protein levels were expressed relative to the control treatment

**FIGURE 3:**
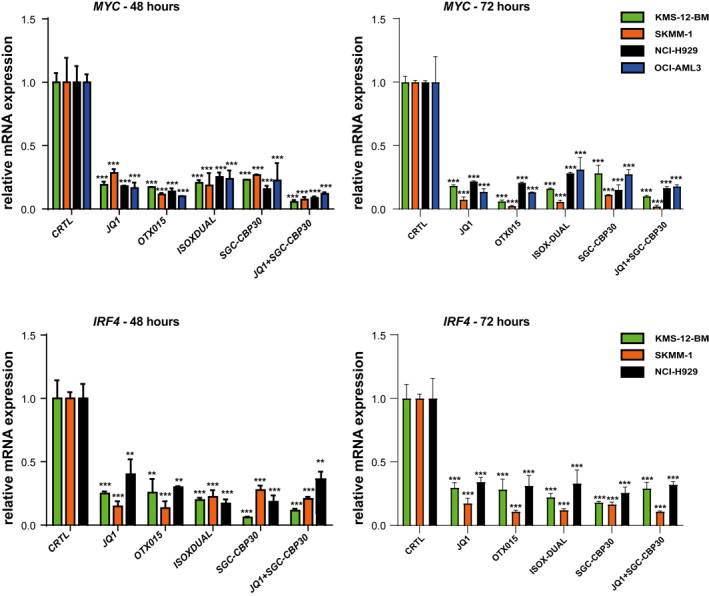
IRF4 and MYC mRNA expression in MM cell‐lines following treatment with JQ1, OTX015, SGC‐CBP30, ISOX‐DUAL and JQ1+ SGC‐CBP30. IRF4 and MYC mRNA expression was analyzed by qPCR following IC_50_ drug treatments for 48 and 72 h in KMS‐12‐BM (green bars), SKMM‐1 (orange bars), NCI‐H929 (black bars) and OCI‐AML3 (blue bars) cells. The control (CTRL) is 2 mM DMSO treatment. Transcript levels were normalized against *β*‐actin expression and expressed relative to the control treatment. Data are shown as mean ± SEM. A *t*‐test was performed with reference to the control. *p < 0.05, **p < 0.01, ***p < 0.001

We next investigated the effects of bromodomain inhibitors on the mRNA and protein expression levels of IRF4 and MYC. We treated the cells with a concentration of drugs at their IC_50_ value (as in Figure 1). As shown by western blotting analysis, we observed a dramatic decrease in the level of MYC protein, following treatment for 4, 8, 24 h (Fig.S2) with a complete abrogation after 48 and 72 h (Figure 2) However, drug treatments did not have a similar effect on IRF4 protein levels. No reduction in IRF4 protein levels was observed at any of the time points when using JQ1 or OTX015 and a slight reduction in IRF4 protein expression (up to 30%) was only observed across all MM cell‐lines when a combination JQ1+SGC‐CBP30 was used (Figure 2, Figure S2). We next examined the effect of drug treatment on the levels of *IRF4* and *MYC* mRNA. Treatment with all drugs significantly decreased both *IRF4* and *MYC* mRNA expression in all cell‐lines after 4, 8, 24, 48 and 72 h (Figure 3, Figure S3), although the mean reduction for *MYC* was more pronounced than that for *IRF4*. In summary, our data show that bromodomain inhibitors effectively reduce *MYC* and *IRF4* mRNA levels and MYC protein levels, but do not show a corresponding effect on IRF4 protein levels.

### 3.3 Bromodomain inhibitors affect the gene‐expression levels of target genes of MYC but not IRF4

**FIGURE 4:**
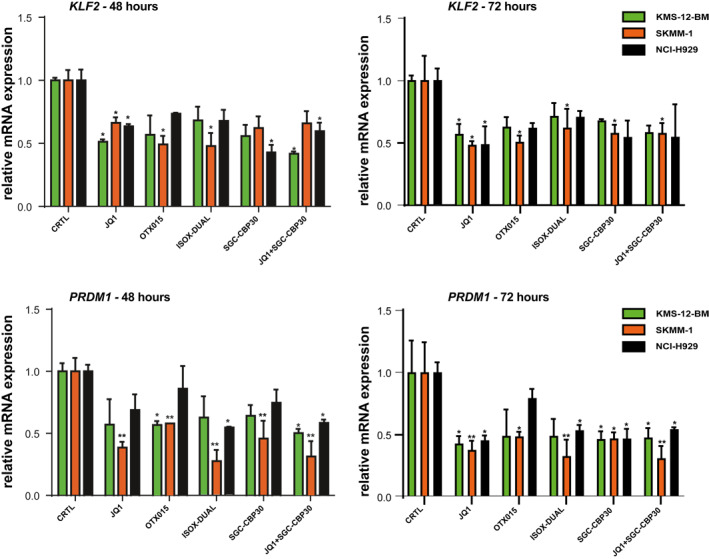
IRF4 downstream gene mRNA expression in MM cell‐lines following treatment with JQ1, OTX015, SGC‐CBP30, ISOX‐DUAL and JQ1+ SGC‐CBP30. KLF2 and PRDM1mRNA expression was analyzed by qPCR following IC_50_ drug treatments for 48 and 72 h in KMS‐12‐BM (green bars), SKMM‐1 (orange bars), and NCI‐H929 (black bars) cells. The control (CTRL) is 2 mM DMSO treatment. Transcript levels were normalized against *β*‐actin expression and expressed relative to the control treatment. Data are shown as mean ± SEM. A *t*‐test was performed with reference to the control. *p < 0.05, **p < 0.01, ***p < 0.001

As protein levels of MYC and IRF4 were unequally affected by drug treatment, we hypothesized that expression of their downstream target genes would also be differentially affected. To test this hypothesis, we measured the impact of drug treatment on the mRNA levels of IRF4 (*KLF2* and *PRDM1)* and MYC (*CDK4* and *hTERT*) downstream targets. We treated the cells with a concentration of drugs corresponding to their IC_50_ value for 4, 8, 24, 48 and 72 h (Figure 4, Figure S4 and Figure S5). At the early time points of 4, 8 and 24 h, no significant reduction of mRNA levels could be detected in the MM cell‐lines for IRF4 downstream target *KLF2* (Fig.S4), whilst a 30% reduction could be seen after 48 and 72 h (Figure 4). A similar trend was observed for *PRDM1* mRNA levels, with small decreases at early time points (Fig.S4) and more substantial decreases of about 50% only occurring after 48 and 72 h (Figure 4).

**FIGURE 5:**
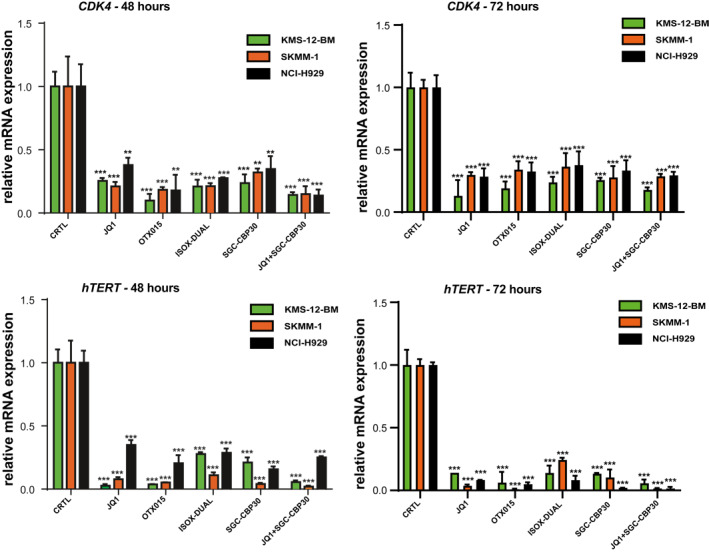
MYC downstream gene mRNA expression in MM cell‐lines following treatment with JQ1, OTX015, SGC‐CBP30, ISOX‐DUAL and JQ1+ SGC‐CBP30. CDK4 and hTERT mRNA expression was analyzed by qPCR following IC_50_ drug treatments for 48 and 72 h in KMS‐12‐BM (green bars), SKMM‐1 (orange bars), and NCI‐H929 (black bars) cells. The control (CTRL) is 2 mM DMSO treatment. Transcript levels were normalized against *β*‐actin expression and expressed relative to the control treatment. Data are shown as mean ± SEM. A *t*‐test was performed with reference to the control. *p < 0.05, **p < 0.01, ***p < 0.001

In contrast, mRNA expression of the MYC downstream targets *hTERT* and *CDK4* were rapidly and effectively decreased by drug treatment in all cell‐lines (Figure 5, Figure S5).

In summary, these results confirm our hypothesis that MYC, but not IRF4 downstream target genes are substantially downregulated as a result of bromodomain inhibition.

### 3.4 Gene and protein network modeling are consistent with a long IRF4 protein half‐life

Given the known feedback loop between MYC and IRF4 in MM cells we asked whether the reduction in IRF4 mRNA, but not protein expression could be explained by the stability of IRF4 protein.

To test this hypothesis and to assess whether the protein and mRNA levels for MYC, IRF4 and their downstream targets following drug exposure were consistent with those expected from the IRF4‐MYC auto‐regulatory loop model, we used computational techniques to model the MYC and IRF4 gene and protein network in MM cells. Computational modeled time courses of PRDM1, IRF4, and MYC protein and mRNA levels were generated by simulating the effect of inhibiting MYC mRNA transcription. In order to compare computational simulations with measured protein and mRNA levels, both experimental and simulated results were normalized to the first timepoint to give a fold change over time.

**FIGURE 6:**
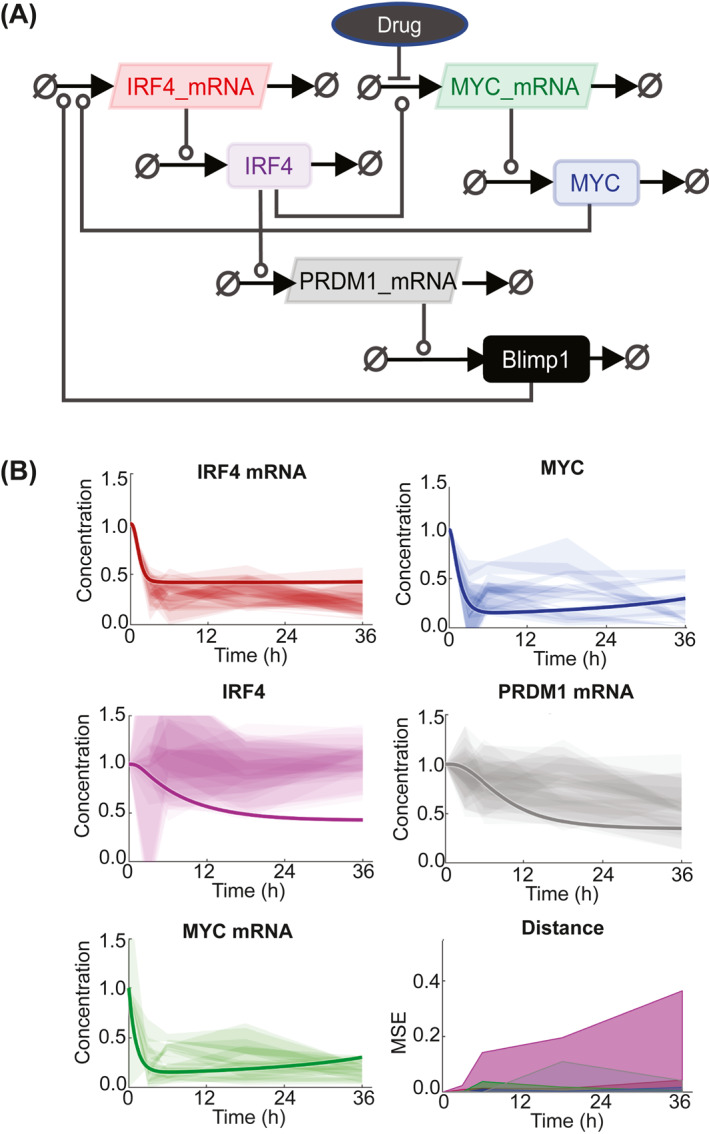
Computational model of the molecular regulatory network in MM cells. A, Systems Biology Graphical Notation (SBGBN) diagram of the model of IRF4, MYC and PRDM1 regulation. Positive regulation is indicated by lines capped with circles. Negative regulation is indicated by lines capped with bars. B, Experimentally measured expression of the indicated molecular species in H929, SKMM‐1, KMS cell‐lines exposed to SGC‐CBP30, JQ1, OTX015, ISOX‐DUAL, and JQ1+SGC‐CBP30 combination. Each shaded region represents the standard deviation of 3 experimental replicates. The modeled response is shown with a solid line. The model assumes a half‐life for IRF4 of 7 h. The squared distance between the mean experimental result and modeled response for each timepoint is shown in the bottom right with colors consistent with other panels

**FIGURE 7:**
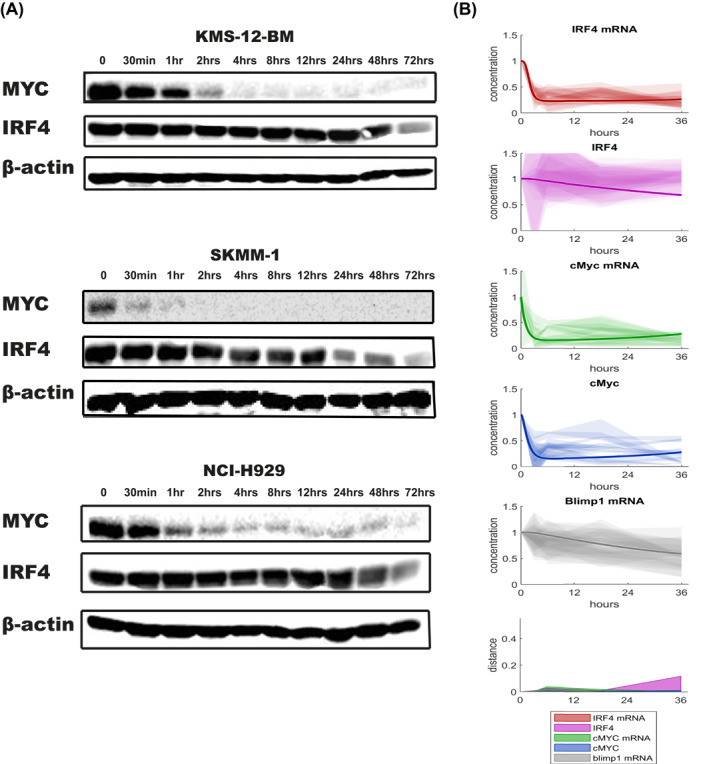
Analysis of IRF4 stability in MM cell‐lines and updated computational model of the molecular regulatory network in MM cell. A, KMS‐12‐BM, SKMM‐1, NCI‐H929 were incubated with 10 μg/ml cycloheximide for the indicated time points and cell lysates analyzed by Western blotting for protein levels of IRF4 and MYC. *β*‐actin was used as a loading control. B, Experimentally measured expression of the indicated molecular species in H929, SKMM‐1, KMS cell‐lines exposed to SGC‐CBP30, JQ1, OTX015, ISOX‐DUAL, and JQ1+SGC‐CBP30 combination. Each shaded region represents the standard deviation of 3 experimental replicates. The modeled response is shown with a solid line. The model uses the experimentally determined IRF4 half‐life. The squared distance between the mean experimental result and modeled response for each timepoint is shown in the bottom right with colors consistent with other panels

As the results are independent from the drug and cell line used, we initially modeled our response based on drugs inhibiting MYC expression (Figure 6A) using the published half‐life for MYC of 30 min^20^ and an estimated of 7 h for IRF4 (no data was found). The squared distance between the mean experimental result and modeled response for each timepoint shows a discrepancy, specifically for IRF4 protein and PRDM1 mRNA levels (Figure 6B), suggesting that IRF4 has a half‐life significantly longer than 7 h. To measure IRF4 protein half‐life, we treated MM cell‐lines with 10 μg/ml cycloheximide to block protein synthesis for up to 72 h and monitored the effect on existing protein levels by western blotting (Figure 7A). We found that IRF4 protein levels decreased slowly in all MM cell‐lines and the half‐life was determine to be 61, 52 and 33 h in KMS‐12‐BM, NCI‐H929 and SKMM‐1 respectively. In contrast to the stability of IRF4, levels of MYC decreased within 30 min in all MM cell‐lines, (half‐lives of 1 hr, 22 and 30 min respectively), in line with published reports.^20^ To test whether a half‐life of 48 h for IRF4 can explain the observed response to the drug we modeled MYC and IRF4 gene and protein network using this longer half‐life. The squared distance between the mean experimental result and modeled response for each timepoint now shows a good agreement between the model and the data (Figure 7B). Despite the overall improvement of the fit, a discrepancy persists for IRF4 protein levels between 24 and 36 h suggesting that the model does not completely recapitulate the data, especially at the later time points.

### 3.5 Gene and protein network modeling suggest that bromodomain inhibitors effects on MM cell‐lines are mainly exerted through MYC transcription repression and not IRF4

**FIGURE 8:**
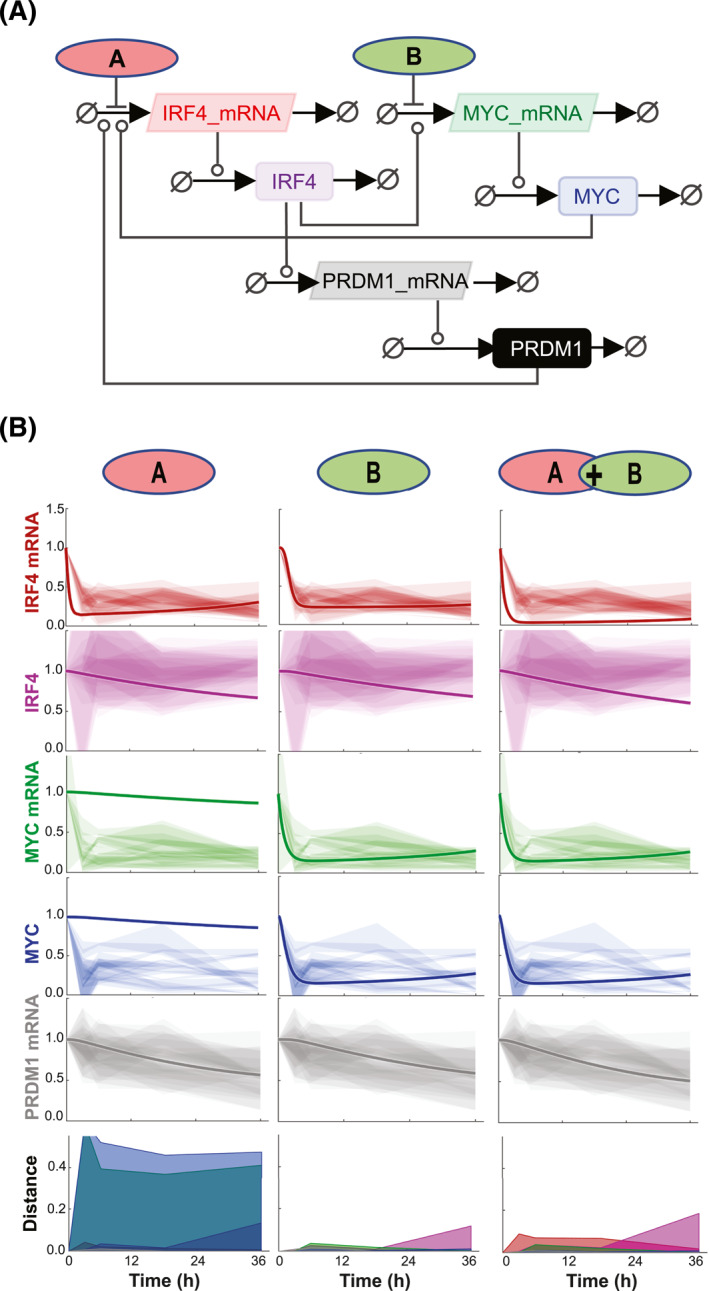
Computational model simulating the effect of a drug acting on *MYC* transcription, *IRF4* transcription or both. Systems Biology Graphical Notation (SBGBN) diagram of the model of IRF4, MYC and PRDM1 regulation. Positive regulation is indicated by lines capped with circles. Negative regulation is indicated by lines capped with bars. Drugs are shown impacting IRF4 transcription (A) and MYC transcription (B). Experimentally measured expression of the indicated molecular species in H929, SKMM‐1, KMS cell‐lines exposed to SGC‐CBP30, JQ1, OTX015, ISOX‐DUAL, and JQ1+SGC‐CBP30 combination. The impact of single targeting IRF4 (A, left) and MYC (B, middle) is shown, along with the combination (A + B, right). Each shaded region represents the standard deviation of 3 experimental replicates. The modeled response is shown with a solid line. The model uses the experimentally determined IRF4 half‐life. The squared distance between the mean experimental result and modeled response for each timepoint is shown in the bottom right with colors consistent with other panels

The initial computational modeling of the predicted drug response on MM cell‐lines was formulated on the assumption of bromodomain inhibition affecting mainly MYC transcription. This was a reasonable assumption based on the observation that unperturbed IRF4 protein levels in MM cell‐lines could be measured following most drug treatment. However, because of a small (30%) but consistent reduction of IRF4 protein levels in response to treatment with the JQ1+ SGC‐CBP30 combination we then asked whether bromodomain inhibitors work through repression of MYC, IRF4 or both. To do so, we used gene and protein network modeling to simulate the effect of a drug acting on the transcription of MYC, IRF4 or both (Figure 8A) using the measured half‐lives of IRF4 and MYC. When comparing the predicted to the experimentally measured expression of MYC, IRF4 and PRDM1 we could conclude that the main effect of the drugs is predicted to be through disruption of MYC transcription (Figure 8B). The modeled response of the effects of a drug acting only on IRF4 transcription poorly predicts the observed protein and mRNA levels, especially those of MYC. Simulating the effects of a drug treatment targeting both MYC and IRF4 transcription improves the match, but not as well when using a single‐hit to MYC model. However, for all models a discrepancy remains between the measured and modeled levels of IRF4 protein after 24 h, pointing at additional and yet uncovered regulatory interactions within the IRF4 network in MM cells. When extrapolated to MM cells in vivo, our work has important implications for the design of new therapeutic strategies.

## 4 DISCUSSION

In this work we studied the effects on MM cell‐lines of two classes of bromodomain (BET and CBP/Ep300) inhibitors, with putatively distinct transcriptional effects, with the aim to disrupt the oncogenic feedback loop between MYC and IRF4. Specifically, we wanted to evaluate the possibility that the combination of these bromodomain inhibitors would have synergistic impact on the viability of MM cells and on the transcription and protein levels of IRF4 and MYC.

Our data showed that while the two BET inhibitors JQ1 and OTX015 showed the most effective inhibition on cell viability, the CBP/Ep300 inhibitor SGC‐CBP/Ep300 and the dual BET‐CBP/Ep300 inhibitor ISOX‐DUAL caused the least effect. Since the combination JQ1+SGC‐CBP30 has a stronger inhibitory effect on cell viability compared to the dual inhibitor alone this suggests that the limited effect of ISOX‐DUAL is caused by its reduced affinity for BRD4 and CPB/EP300. Our data also indicate that combining JQ1 and SGC‐CBP30 does not lead to synergistic or antagonistic cytotoxic effects on MM cell‐lines. In line with previous studies,^14^, ^15^, ^17^, ^21^ we found that these drugs cause MYC downregulation at protein and mRNA levels. Interestingly, within the time frame and for all inhibitors we have observed largely unaffected levels of IRF4 protein and downstream target gene mRNA levels. Using computational modeling of a network of MM molecular interactions, we could show that these results can be partially explained by the high stability of the IRF4 protein (>48 h). Finally, the modeling data also implies that any effect observed on MM cell‐lines for both inhibitors is not exerted through IRF4 but mainly through MYC. These results are in contrast with previous data^17^ supporting the idea that SGC‐CBP30 treatment on MM cell line causes cell cytotoxicity via targeting of IRF4. However, more recent data show that inhibition of CBP/EP300 bromodomains can interfere with GATA1 and MYC‐driven transcription by displacing CBP/EP300 from GATA1 and MYC binding sites at enhancers leading to a decrease in the level of acetylation of these regulatory regions. This in turn reduces gene‐expression of both GATA1 and MYC.^22^

Our data shows that IRF4 is characterized by a long half‐life in a panel of MM cell‐lines. Previous studies have shown a variability in the half‐life's values for IRF proteins (IRF1∼30 min, IRF7∼5 h, IRF2∼8 h, IRF3∼60 h).^23^, ^24^ The basis of these varied half‐lives is unclear, but it may involve differences in ubiquitin‐mediated degradation through differential in expression of ubiquitin‐specific proteases (USPs). Alterations of USP enzymes are implicated in the pathogenesis of various cancers and USP15 has been reported to be overexpressed in MM cells and inhibit MM apoptosis.^25^, ^26^ Interestingly, USP4 interacts with, stabilizes and deubiquitinates IRF4,^27^ which could be provide an explanation for the long IRF4 half‐life. Further work will be required to determine if these USPs have any role in the regulation of IRF4 stability in MM cells.

A growing body of preclinical and clinical evidence suggests that bromodomain inhibition could be an important therapeutic approach in a number of hematologic malignancies.^28^ Furthermore, in vivo and in vitro evidence suggests synergistic cytotoxicity of bromodomain inhibitors and immunomodulatory drugs (IMiDs) in MM^29^ and primary effusion lymphoma.^30^ lMiDs are known to bind cereblon, which activates E3‐ubiquitin ligase resulting in the degradation of IKZF1 and IKZF3.^31^ Downregulation of IKZF1 and IKZF3 then suppresses IRF4 transcription. Therefore IMiDs, just like bromodomain inhibitors, indirectly inhibit IRF4 expression. Our studies suggest that indirect inhibition of IRF4, either via IMiDs or bromodomain inhibition, might not be effective at interfering with IRF4 and its oncogenic transcription program in MM because of its stability. Future work aimed at targeting the IRF4 addiction in MM may be more effective if re‐focused on direct inhibition or degradation of IRF4, which could be then used in synergistic combination to address relapsed or refractory cases of MM for which presently limited choices exist.

## CONFLICT OF INTEREST

The authors declare that the research was conducted in the absence of any commercial or financial relationships that could be construed as potential conflicts of interest.

### PEER REVIEW

The peer review history for this article is available at https://publons.com/publon/10.1002/hon.3016.

## ETHICS STATEMENT

No ethical approvals were required for the studies conducted in this article.

## Supporting information

Supporting Information S1

Figure S1

Figure S2

Figure S3

Figure S4

Figure S5

## Data Availability

All data needed to evaluate the conclusions in the paper are present in the paper and/or the Supplementary Materials. Additional data related to this paper may be requested from the authors.

## References

[1] Klein U , Casola S , Cattoretti G , et al. Transcription factor IRF4 controls plasma cell differentiation and class‐switch recombination. Nat Immunol. 2006;7(7):773‐782. 10.1038/ni1357 16767092

[2] Agnarelli A , Chevassut T , Mancini EJ . IRF4 in multiple myeloma‐Biology, disease and therapeutic target. Leuk Res. 2018;72:52‐58. 10.1016/j.leukres.2018.07.025 30098518

[3] Wang L , Yao ZQ , Moorman JP , Xu Y , Ning S . Gene expression profiling identifies IRF4‐associated molecular signatures in hematological malignancies. PLoS One. 2014;9(9):e106788. 10.1371/journal.pone.0106788 25207815 PMC4160201

[4] Iida S , Rao PH , Butler M , et al. Deregulation of MUM1/IRF4 by chromosomal translocation in multiple myeloma. Nat Genet. 1997;17(2):226‐230. 10.1038/ng1097-226 9326949

[5] Yoshida S , Nakazawa N , Iida S , et al. Detection of MUM1/IRF4‐IgH fusion in multiple myeloma. Leukemia. 1999;13(11):1812‐1816. 10.1038/sj.leu.2401563 10557056

[6] Shaffer AL , Emre NC , Lamy L , et al. IRF4 addiction in multiple myeloma. Nature. 2008;454(7201):226‐231. 10.1038/nature07064 18568025 PMC2542904

[7] Bai H , Wu S , Wang R , Xu J , Chen L . Bone marrow IRF4 level in multiple myeloma: an indicator of peripheral blood Th17 and disease. Oncotarget. 2017;8(49):85392‐85400. 08/03 03/29/received 07/12/accepted. 10.18632/oncotarget.19907 29156727 PMC5689617

[8] Morelli E , Leone E , Cantafio ME , et al. Selective targeting of IRF4 by synthetic microRNA‐125b‐5p mimics induces anti‐multiple myeloma activity in vitro and in vivo. *Leukemia* . 2015;29(11):2173‐2183. 10.1038/leu.2015.124 PMC4635336 25987254

[9] Rajkumar SV . Multiple myeloma: 2016 update on diagnosis, risk‐stratification, and management. Am J Hematol. 2016;91(7):719‐734. 10.1002/ajh.24402 27291302 PMC5291298

[10] Dingli D , Ailawadhi S , Bergsagel PL , et al. Therapy for relapsed multiple myeloma: guidelines from the mayo stratification for myeloma and risk‐adapted therapy. Mayo Clin Proc. 2017;92(4):578‐598. 10.1016/j.mayocp.2017.01.003 28291589 PMC5554888

[11] Chng WJ , Huang GF , Chung TH , et al. Clinical and biological implications of MYC activation: a common difference between MGUS and newly diagnosed multiple myeloma. Leukemia. 2011;25(6):1026‐1035. 10.1038/leu.2011.53 21468039 PMC3432644

[12] Lin Y , Wong K , Calame K . Repression of c‐myc transcription by Blimp‐1, an inducer of terminal B cell differentiation. Science. 1997;276(5312):596‐599. 10.1126/science.276.5312.596 9110979

[13] Chaidos A , Caputo V , Karadimitris A . Inhibition of bromodomain and extra‐terminal proteins (BET) as a potential therapeutic approach in haematological malignancies: emerging preclinical and clinical evidence. Therapeutic Adv Hematol. 2015;6(3):128‐141. 10.1177/2040620715576662 PMC4480520 26137204

[14] Delmore JE , Issa GC , Lemieux ME , et al. BET bromodomain inhibition as a therapeutic strategy to target c‐Myc. Cell. 2011;146(6):904‐917. 10.1016/j.cell.2011.08.017 21889194 PMC3187920

[15] Shi J , Song S , Han H , et al. Potent activity of the bromodomain inhibitor OTX015 in multiple myeloma. Mol Pharm. 2018;15(9):4139‐4147. 10.1021/acs.molpharmaceut.8b00554 30048594

[16] Ogryzko VV , Schiltz RL , Russanova V , Howard BH , Nakatani Y . The transcriptional coactivators p300 and CBP are histone acetyltransferases. Cell. 1996;87(5):953‐959. 10.1016/s0092-8674(00)82001-2 8945521

[17] Conery AR , Centore RC , Neiss A , et al. Bromodomain inhibition of the transcriptional coactivators CBP/EP300 as a therapeutic strategy to target the IRF4 network in multiple myeloma. Elife. 2016;5. 10.7554/eLife.10483 PMC4775225 26731516

[18] Mitchell S , Mercado EL , Adelaja A , et al. An NFκB activity calculator to delineate signaling crosstalk: type I and II interferons enhance NFκB via distinct mechanisms. Front Immunol. 2019;10:1425. 10.3389/fimmu.2019.01425 31293585 PMC6604663

[19] Chekler EL , Pellegrino JA , Lanz TA , et al. Transcriptional profiling of a selective CREB binding protein bromodomain inhibitor highlights therapeutic opportunities. Chem Biol. 2015;22(12):1588‐1596. 10.1016/j.chembiol.2015.10.013 26670081

[20] Hann SR , Eisenman RN . Proteins encoded by the human c‐myc oncogene: differential expression in neoplastic cells. Mol Cell Biol. 1984;4(11):2486‐2497. 10.1128/mcb.4.11.2486 6513926 PMC369080

[21] Mertz JA , Conery AR , Bryant BM , et al. Targeting MYC dependence in cancer by inhibiting BET bromodomains. Proc Natl Acad Sci U. S. A. 2011;108(40):16669‐16674. 10.1073/pnas.1108190108 21949397 PMC3189078

[22] Garcia‐Carpizo V , Ruiz‐Llorente S , Sarmentero J , Grana‐Castro O , Pisano DG , Barrero MJ . CREBBP/EP300 bromodomains are critical to sustain the GATA1/MYC regulatory axis in proliferation. Epigenetics Chromatin. 2018;11(1):30. 10.1186/s13072-018-0197-x 29884215 PMC5992658

[23] Watanabe N , Sakakibara J , Hovanessian AG , Taniguchi T , Fujita T . Activation of IFN‐beta element by IRF‐1 requires a posttranslational event in addition to IRF‐1 synthesis. Nucleic Acids Res. 1991;19(16):4421‐4428. 10.1093/nar/19.16.4421 1886766 PMC328629

[24] Prakash A , Levy DE . Regulation of IRF7 through cell type‐specific protein stability. Biochem Biophys Res Commun. 2006;342(1):50‐56. 10.1016/j.bbrc.2006.01.122 16472772 PMC1647301

[25] Zhou L , Jiang H , Du J , et al. USP15 inhibits multiple myeloma cell apoptosis through activating a feedback loop with the transcription factor NF‐κBp65. Exp Mol Med. 2018;50(11):151. 10.1038/s12276-018-0180-4 PMC6244212 30459344

[26] Wei R , Liu X , Yu W , et al. Deubiquitinases in cancer. Oncotarget. 2015;6(15):12872‐12889. 10.18632/oncotarget.3671 25972356 PMC4536986

[27] Guo Z , Xu P , Ge S , et al. Ubiquitin specific peptidase 4 stabilizes interferon regulatory factor protein and promotes its function to facilitate interleukin‐4 expression in T helper type 2 cells. Int J Mol Med. 2017;40(4):979‐986. 10.3892/ijmm.2017.3087 28791349 PMC5593473

[28] Abedin SM , Boddy CS , Munshi HG . BET inhibitors in the treatment of hematologic malignancies: current insights and future prospects. Onco Targets Ther. 2016;9:5943‐5953. 10.2147/OTT.S100515 27729803 PMC5047722

[29] Diaz T , Rodriguez V , Lozano E , et al. The BET bromodomain inhibitor CPI203 improves lenalidomide and dexamethasone activity in in vitro and in vivo models of multiple myeloma by blockade of Ikaros and MYC signaling. Haematologica. 2017;102(10):1776‐1784. 10.3324/haematol.2017.164632 28751557 PMC5622862

[30] Gopalakrishnan R , Matta H , Tolani B , Triche T, Jr. , Chaudhary PM . Immunomodulatory drugs target IKZF1‐IRF4‐MYC axis in primary effusion lymphoma in a cereblon‐dependent manner and display synergistic cytotoxicity with BRD4 inhibitors. Oncogene. 2016;35(14):1797‐1810. 10.1038/onc.2015.245 26119939 PMC4486341

[31] Lu G , Middleton RE , Sun H , et al. The myeloma drug lenalidomide promotes the cereblon‐dependent destruction of Ikaros proteins. Science. 2014;343(6168):305‐309. 10.1126/science.1244917 24292623 PMC4070318

