# Supplementary material for: Dissecting the impact of bromodomain inhibitors on the Interferon Regulatory Factor 4‐MYC oncogenic axis in multiple myeloma
Source: Hematol Oncol. 2022 May 18;40(3):417–29. doi: 10.1002/hon.3016 (PMC9543246; doi:10.1002/hon.3016)
Supplement: Supplementary file 1 — Supporting Information S1 [file HON-40-417-s002.pdf]

Supplementary Methods

Cell lines

Multiple Myeloma cell lines KMS-12-BM, NCI-H929, SKMM-1 and acute myeloid leukemia OCI-AML3 were grown at 37°C in a humidified environment in 5% CO_2 as follows: KMS-12-BM, SKMM-1 and OCI-AML3 were grown in RPMI medium with 20% FBS, 1% Penicillin-Streptomycin-L-glutamine, 1% Sodium-Pyruvate and 0.05% Thioglycerol. NCI-H929 cells were grown in RPMI medium with 20% FBS, 1% Penicillin Streptomycin-L-glutamine, 1% Sodium Pyruvate, 0.05% β-mercaptoethanol. Cells were split twice-weekly at $0.5x10^6$ cells/ml.

Cell viability assay and inhibitor studies

Cells were plated at 20,000 cells per well in 96 well plates and treated with 2mM DMSO or indicated compounds for 48 h and 72 h at 37°C (full concentration range used 0.01-10µM). Cell viability was analysed by performing CellTiter-Blue® Cell Viability Assay following manufacturer's instructions (Promega). After 48 h and 72 h treatment, 20µL CellTiter-Blue® Reagent (Promega G8080) was added to each 96 wells plate. The plates were shaken for 10 s and then incubated for 2 h at 37°C. After 2 h the plates were shaken on an orbital shaker for 10 s and fluorescence was recorded at 560/590nm. Cells were plated in triplicate/per condition and assays were performed at least 3 times. The IC50 was determined using nonlinear regression (curve fit) with log (inhibitor) vs response Variable slope (four parameters) using GraphPad Prism Version 6.01. The mean IC50 was determined and statistical significance was performed by one-way ANOVA followed by Tukey's test to compare statistical differences among IC50s of different cell lines treated with a specific drug.

Statistical analysis

Statistical analysis was carried out using Statgraphics (version XVI) and GraphPad Prism (version 6.01) software. Data tabulation and descriptive statistics were performed by using Excel program (Office 2016). Data are expressed as a mean of three independent experiments with three replicates. Error bars represent standard error of the mean (SEM). Normality was tested by Shapiro-Wilk and Kolmogorov-Smirnov tests. Homoscedasticity was tested by Levene's test. For multiple comparisons of normally distributed data, one-way ANOVA analysis of variance with the Tukey's HSD post-hoc test was performed. P-values < 0.05 were considered to be statistically significant.

Western Blotting

Cell lysates were prepared by adding 100μL 1x Gel Sample Buffer (50mM Tris pH 6.8, 4% SDS, 5% β-Mercaptoethanol, 0.01% Bromophenol blue, 10% Glycerol, 1mM EDTA) per 10^6 cells. The lysates were then sonicated at 25% 5x 10 s with 10 s gaps on ice using sonics Vibra-Cell™. 10 % acrylamide gel was prepared. Samples were separated by SDS-PAGE using 10 % acrylamide gel for 90 min at 120V. Proteins were transferred on to Protran nitrocellulose membranes (Schleicher and Schuell) for 90 min at 85 V. Membranes were blocked using 5% milk in PBS-Tween 0.5% for 1 h. Membranes were incubated with primary antibodies (see below) overnight at 4 °C. Membranes were then washed 3x10 min in PBS-Tween and then incubated with HRP-conjugated secondary antibodies (see below) for 1 h at room temperature. Membranes were then washed 3x10 min in PBS-Tween and Pierce™ ECL western blotting substrate (Thermo Fisher Scientific 32209) added to the membrane for visualization at LI-COR machine. Stripping was performed by adding Tween® 20 [BP337-100, Thermo Fisher] to the membranes for 10 min. Membranes were then

washed 3x10 min in PBS-Tween and blocked with 5% milk in PBS-Tween for 1 h. The following primary and secondary antibodies were added as described above. IRF4 (1:10000, Anti-MUM1 antibody [EP5699] (ab133590), Abcam), MYC (1:300, Antibody (9E10): sc-40, Santa Cruz Biotechnology) and β-actin (1:5000, Anti-Actin antibody A2066, Sigma-Aldrich). HRP-conjugated secondary antibodies used: anti-rabbit (1:3000, Abcam ab205718) and anti-mouse (1:5000, Cell signalling 7076S). Western blots were performed in triplicate.

RNA extraction, cDNA synthesis, and quantitative RT-PCR

Total RNA was extracted using Monarch total RNA miniprep kit (T2010S). RNA concentrations were determined using a NanoDrop 2000 instrument (Thermo Scientific). cDNA was synthesized by using ImProm-II™ Reverse Transcription System kit with random primers (Promega A3800). RNA/primer mixes were prepared on ice with 1µg RNA, 1µl random primers and Nuclease-Free Water up to 5µL. Reverse transcription reaction contained 4.0µL ImProm-II™ 5X Reaction Buffer, 4µL $MgCl_2$ (6.6mM), 1.0µL dNTP Mix, 0.5µL Recombinant RNasin® Ribonuclease Inhibitor, 1.0µL ImProm-II™ Reverse Transcriptase and Nuclease-Free Water up to 15µL. 15µl of Reverse transcription reaction were then mixed with 5µL RNA/primer mixes in PCR tubes (Axygen® PCR-02-C). cDNA was synthesized by placing the PCR tubes first in a controlled-temperature heat block at 25°C for 5 min for the annealing reaction, then in a controlled-temperature heat block at 42°C for 1 h for the extension reaction. The reverse transcriptase was then inactivated incubating the reaction tubes in a controlled-temperature heat block at 72°C for 15 min. cDNA was then used for PCR amplification.

Real time PCR was performed using an Applied Biosystems StepOnePlus PCR machine. In the real time PCR reactions cDNAs represents 20% of the reaction volume. For each sample we used a reaction volume of 15µL that was composed by 1X of GoTaq® qPCR Master mix, 2X (Promega A6002), 0.15µM of each primer, Nuclease-Free Water and 3µL cDNA. cDNA was amplified by heating samples to 95°C for 10 min followed by 40 cycles of 95°C for 15 s and 60°C for 1 minute prior to dissociation curve analysis. Serial dilutions of cDNA were used to generate standard curves for each primer set. Quantitative RT-PCR reactions were performed in triplicate.

Primer sequences used for Real time PCR: *IRF4* Fw 5'-AACAAACTGGAGAGAGACCAGACC-3' Rv 5'-CCTCTCCAAAGCATAGAGTCACC-3'; *MYC* Fw 5'-CCTGGTGCTCCATGAGGAGAC-3' Rv 5'-CAGACTCTGACCTTTTGCCAGG-3'; *PRDM1* Fw 5'-TACATACCAAAGGGCACACG-3' Rv 5'- TGAAGCTCCCCTCTGGAATA-3'; *KLF2* Fw 5'-AGACCTACACCAAGAGTTCGCATC-3' Rv 5'- CATGTGCCGTTTCATGTGCAGC-3'; *CDK4* Fw 5'-CTTCTGCAGTCCACATATGCAACA-3' Rv 5'-CAACTGGTCGGCTTCAGAGTTTC-3'; *hTERT* Fw 5'-GGAGCAAGTTGCAAAGCATTG-3' Rv 5'-TCCCACGACGTAGTCCATGTT-3'; *β-actin* Fw 5'-TTCTACAATGAGCTGCGTGTG-3' Rv 5'-GGGGTGTTGAAGGTCTCAAA-3'.

Protein half-life

Cells were grown in in T25 cm^2 flasks and incubated with cycloheximide for up to 72 h. After 72 h cells were pipetted into 50mL tubes and pelleted at 378 g for 10 min at 4°C. The media was aspirated off and cells resuspended in PBS. An aliquot of cells

was removed to count the total cell number. Cells were re-pelleted (378 g for 10 min at 4°C) and 1mL/5x10^6 cells of PBS was added to each tube, followed by a final spin at 20000 g for 10-20 s (pulse). PBS was aspirated off and the pellet was frozen on dry ice. Western blotting analysis was then performed. The half-life protein was quantified by using nonlinear regression (curve fit) with one phase decay GraphPad Prism Version 6.01. The experiment was performed in triplicate.

Gene and protein network modelling

Each modelled molecular species is modelled as having an expression and a degradation term, such that:

$$\frac{d[X]}{dt} = Expression - Degradation$$

Expression and degradation terms were assumed constant unless influenced by regulation as indicated by promotion or inhibition lines in the diagram (Fig.6). Drugs are modelled by dividing expression by the drug's activity such that:

$$Expression(t) = ExpressionWithoutDrug(t)/drug(t)$$

The drug's activity was assumed to rapidly increase within 15 min and then slowly decay over 48 h.
