# Supplementary figures and images for: Dissecting the impact of bromodomain inhibitors on the Interferon Regulatory Factor 4‐MYC oncogenic axis in multiple myeloma
Source: Hematol Oncol. 2022 May 18;40(3):417–29. doi: 10.1002/hon.3016 (PMC9543246; doi:10.1002/hon.3016)

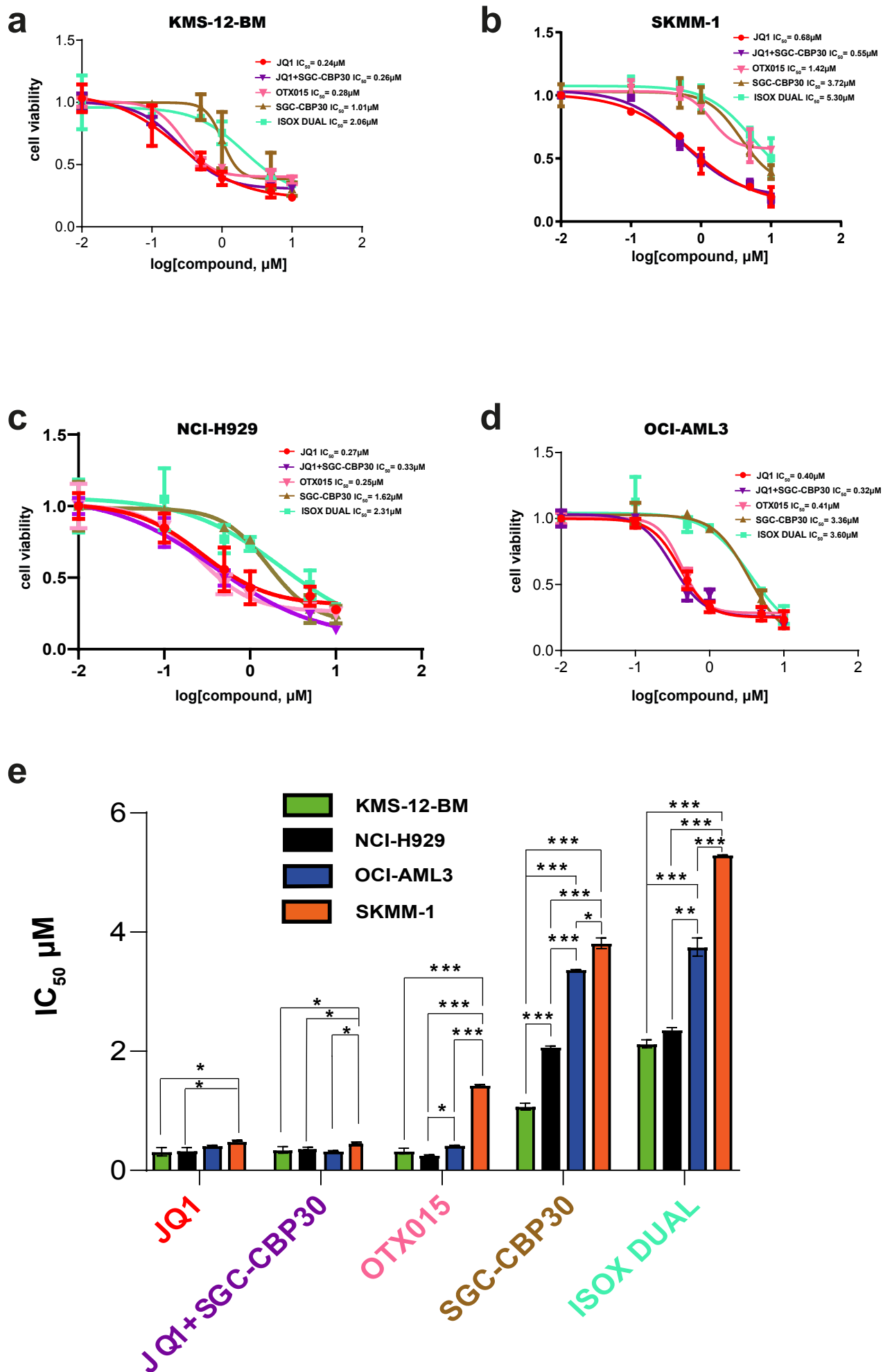

Supplementary Fig.1

Supplement: Supplementary file 2 — Figure S1 [file HON-40-417-s006.pdf]

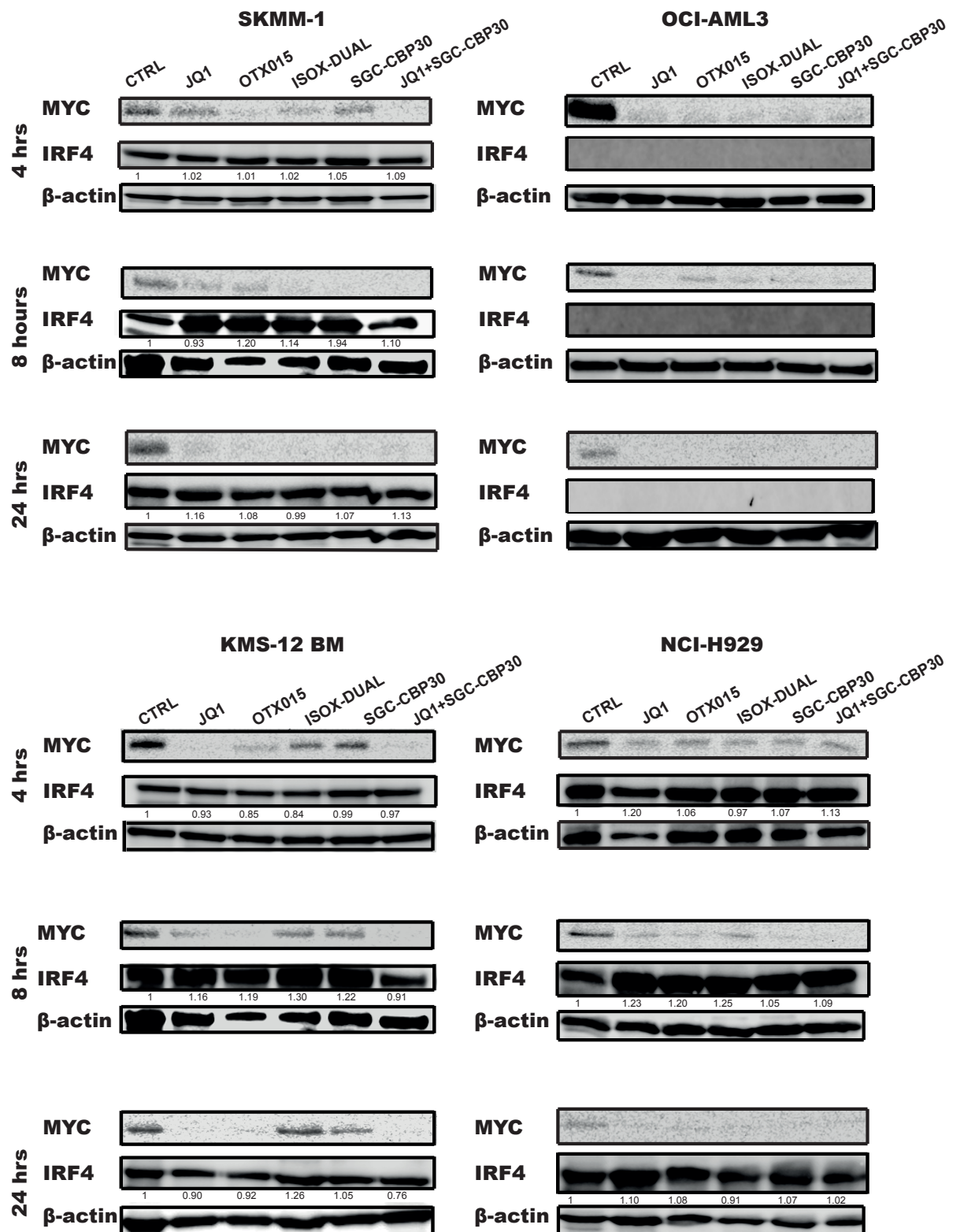

Supplementary Fig.2

Supplement: Supplementary file 3 — Figure S2 [file HON-40-417-s003.pdf]

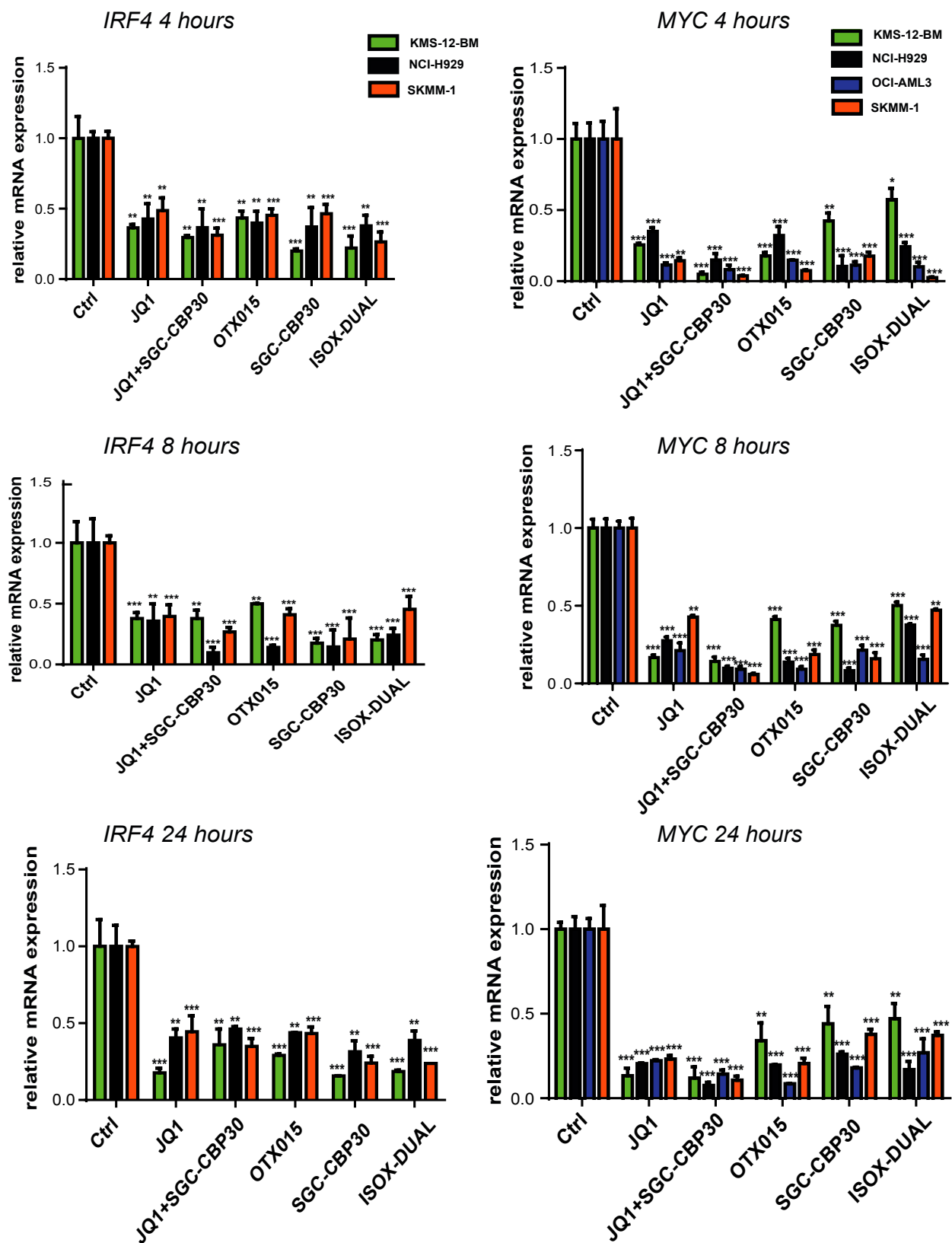

**Supplementary Fig.3**

Supplement: Supplementary file 4 — Figure S3 [file HON-40-417-s005.pdf]

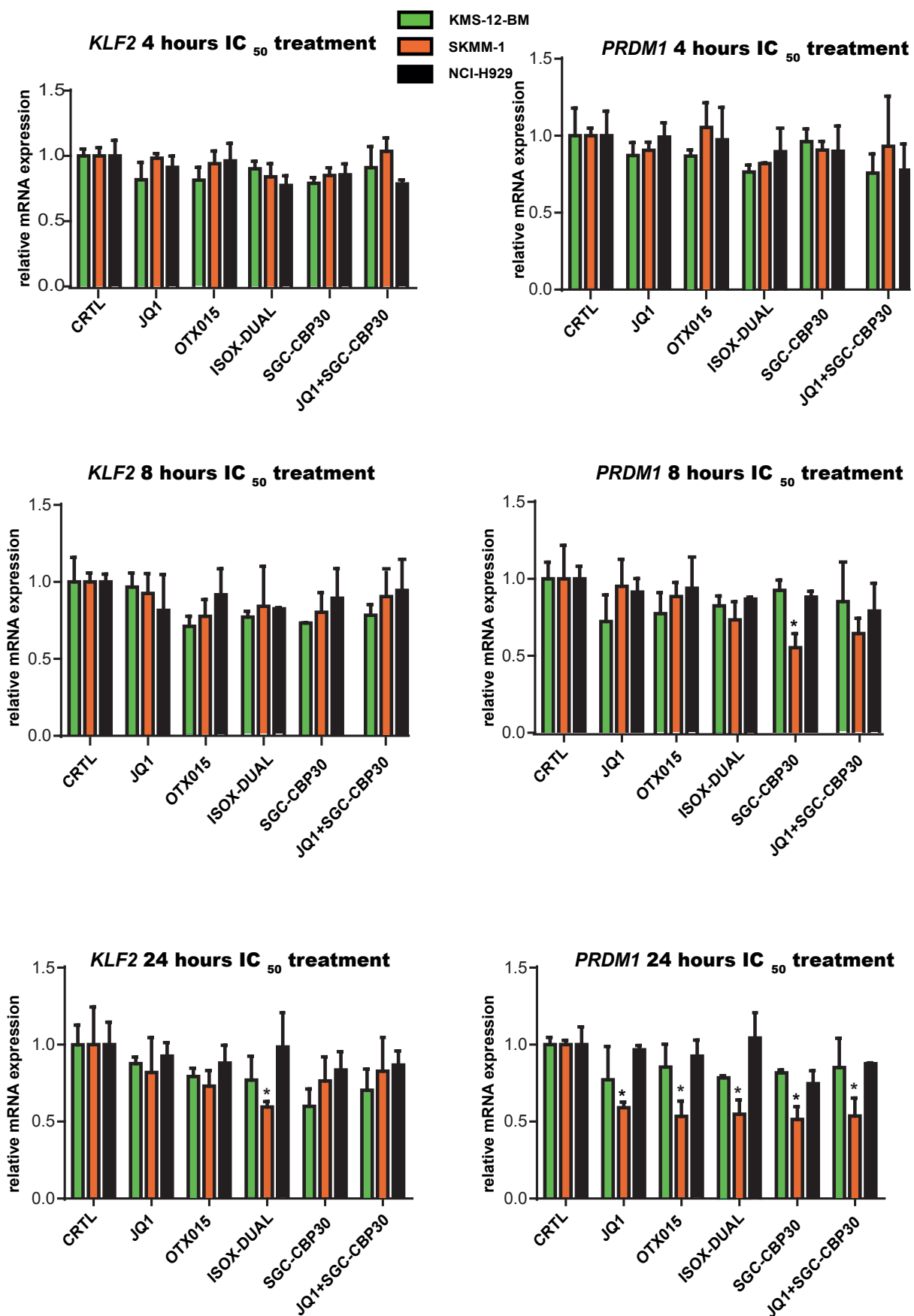

Supplementary Fig.4

Supplement: Supplementary file 5 — Figure S4 [file HON-40-417-s004.pdf]

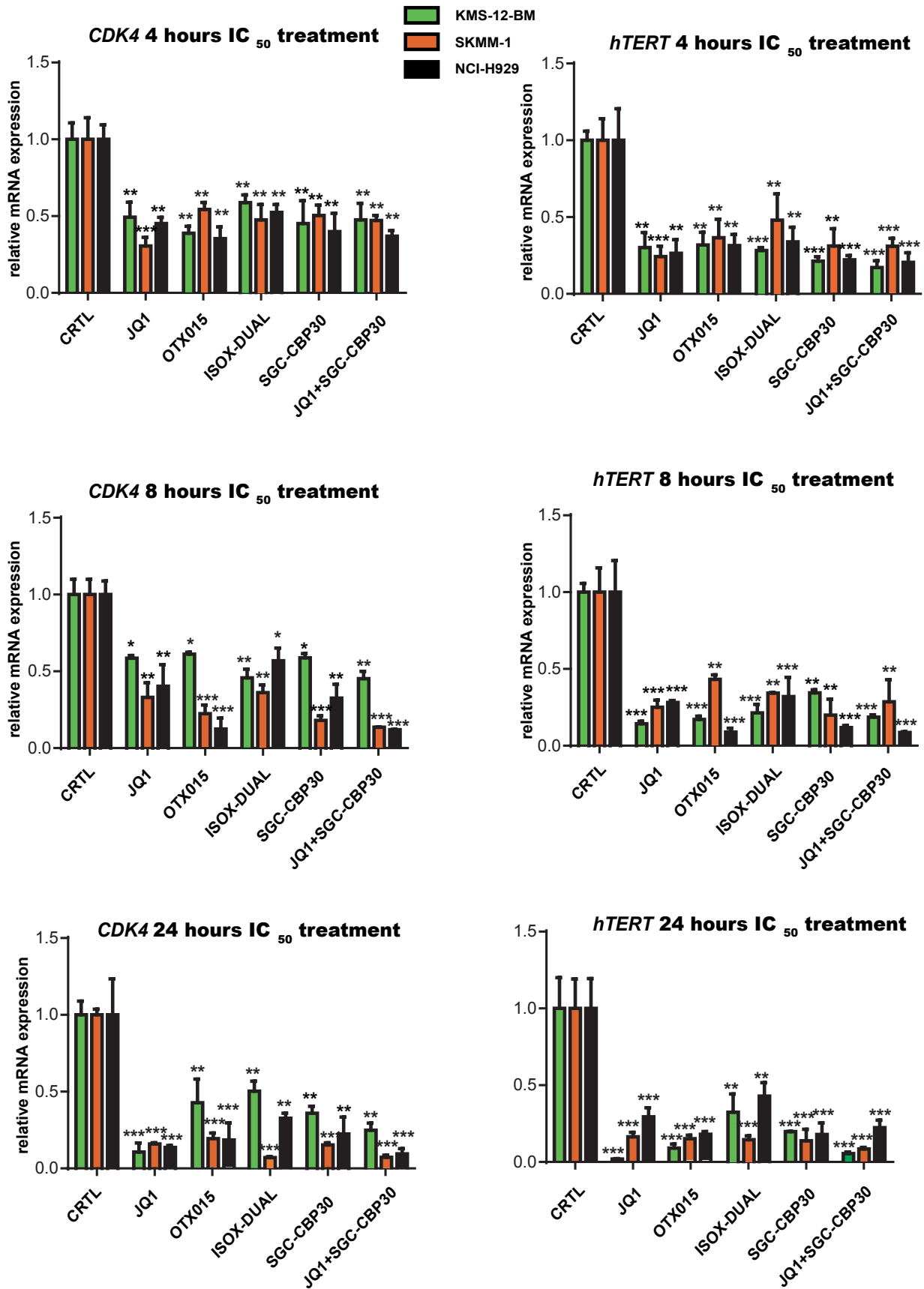

**Supplementary Fig.5**

Supplement: Supplementary file 6 — Figure S5 [file HON-40-417-s001.pdf]
